# Etiology profile of the patients implanted in the cochlear implant program

**DOI:** 10.1590/S1808-86942011000100003

**Published:** 2015-10-19

**Authors:** Clara Maria Dias Ferreira Calháu, Luiz Rodolpho Penna Lima Júnior, Ana Maria da Costa dos Santos Reis, Ana Karla Bigois Capistrano, Danielle do Vale Silva Penna Lima, Ana Carolina Dias Ferreira Calháu, Fábio de Alencar Rodrigues Júnior

**Affiliations:** 1 ENT. Full Professor of Otolaryngology - Universidade Potiguar; 2 ENT. Head of the Cochlear Implant Program in Natal; 3 Speech and Hearing Therapist. MBA - Universidade Potiguar, Head of the Speech and Hearing Program - Universidade Potiguar; 4 Speech and Hearing Therapist. Expert in Audiology and Oral Movement. Professor of Audiology - Universidade Potiguar; 5 Speech and Hearing Therapist. Audiologist - Cochler Implant Program in Natal; 6 Medical Student -Universidade Potiguar; 7 Medical Student - Universidade Potiguar

**Keywords:** diagnosis, hearing loss, cochlear Implantation

## Abstract

A to investigate the major etiological agents that caused deafness in the studied population is of great relevance to prognostic and treatment purposes and it serves as sampling for future actions in the public health.

**Aim:** to check the different hearing impairment etiologies of patients in the cochlear implant program; we studied the etiologies described in order to correlate etiology with age.

**Materials and Methods:** Longitudinal historical cohort study which analyzed 200 charts from patients submitted to cochlear implantation in the program between August of 2000 and May of 2008. Collected data: age; gender; state of birth; hearing impairment etiology.

**Results and conclusion:** Unknown etiology prevailed as main cause, and this indicates the need to continue carrying out genetic studies in those cases of congenital sensorineural hearing loss without an apparent cause in order to trace and etiological profile. Rubella was the second most found cause, and for this etiology there already are preventive measures as there are for meningitis. Even then, the incidences of these diseases remain high. In the correlation of the different etiologies and age ranges, we noticed varied etiologies when we compared children, young adults, adults and the elderly.

## INTRODUCTION

Hearing impairment is one of the disorders which disable the individual in terms of communication, impacting different aspects of his/her life: emotional, social, psychological and intellectual.

Data from the *American Academy of Pediatrics* (AAP - 1999) state that deafness is present in 1 to 3 of every 100 healthy newborns, and in 2 to 4 of every 100 infants being followed up in the neonatal ICU. On September 28 of 2004, Ordinance 2.073/GM was enacted, contemplating hearing health, stating that hearing impairment is a public health issue which requires government measures to assess, control and care for people with such disorder.

To investigate the main etiological agents which caused hearing impairment in this population studied is of great relevance for treatment prognosis, and serves as sampling material for future public health care actions.

The goal of the present study was to investigate the different hearing impairment etiologies of patients implanted in the Cochlear Implant Program, to survey the etiologies found and correlate etiology with age.

## MATERIALS AND METHODS

We carried out a longitudinal historical cohort, based on the analysis of 200 charts from the patients submitted to cochlear implant surgery between August of 2000 and May of 2008. The data collected were associated with age, gender, state of birth and the very etiology of the hearing impairment. Moreover, we plotted and analyzed this data. The project was approved by the Ethics in Research Committee, CAAE, 0209.0.052.000-09.

## RESULTS

**Figure 1:**
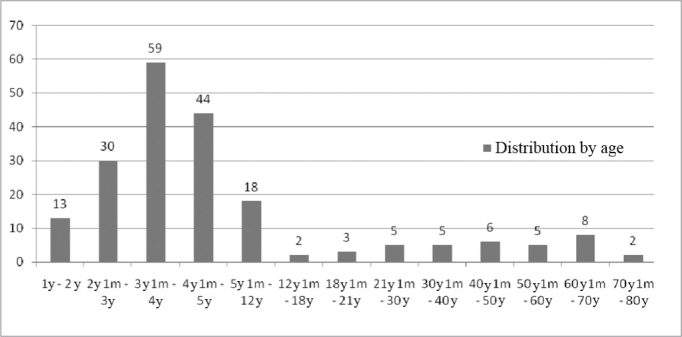
Implanted patients' distribution according to age.

The age range of the patients who took part in this study was between 1 and 73 years, and most of them were between 3 and 4 years, as described in Figure 1.

**Figure 2:**
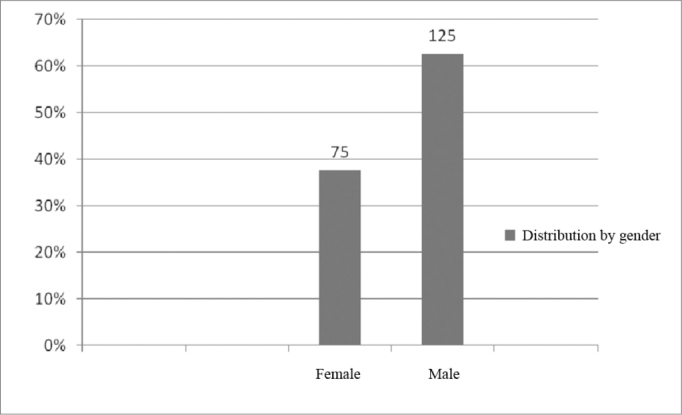
Implanted patients' distribution according to gender.

As to the distribution per gender, there was a prevalence of males (62.5%), as depicted on Figure 2.

**Figure 3:**
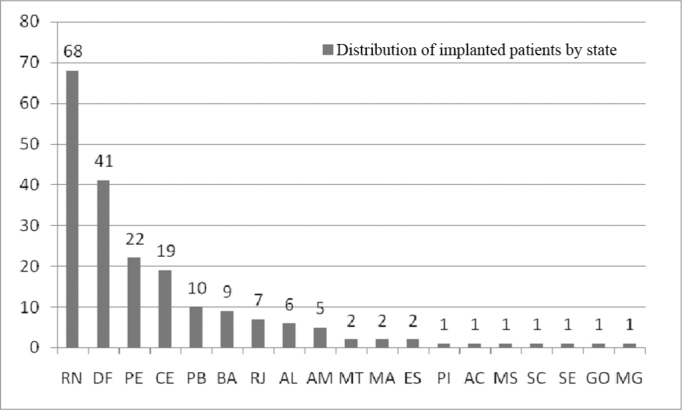
Implanted patients' distribution according to state of birth.

Of the 200 implanted patients, 68 came from Rio Grande do Norte, 41 from Brasília, 22 from Pernambuco and 19 from Ceará. Nonetheless, there were patients from all the regions of the country, as depicted on Fig. 3.

**Figure 4:**
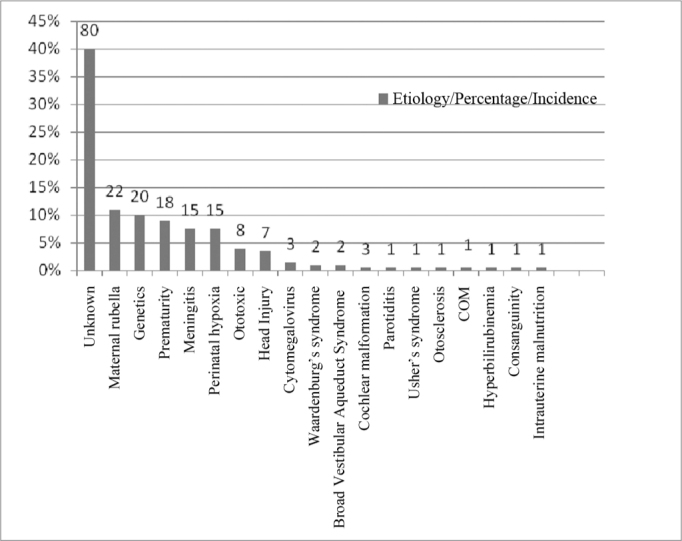
Implanted patients' distribution according to the etiology found.

In the 200 charts assessed, the most commonly found etiology was the unknown type (40%), followed by maternal rubella (11%), genetics (10%) and prematurity (9%), as seen in Fig. 4.

**Figure 5:**
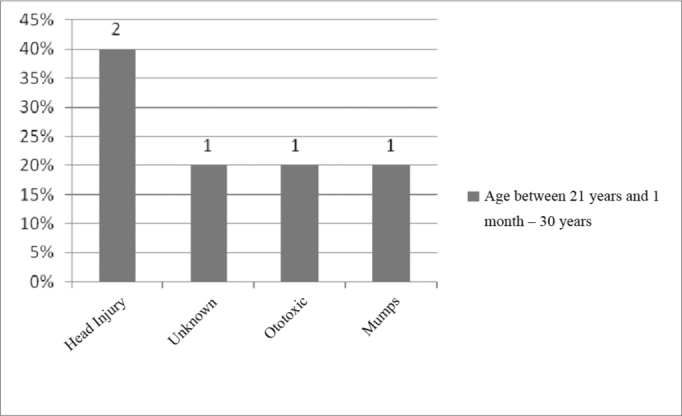
Implanted patient's etiology distribution in the age range between 21 years and 1 month and 30 years.

In the age range between 21 years and 1 months and 30 years of age, Head Injury (HI) was the most commonly found problem, as depicted on Fig. 5.

**Figure 6:**
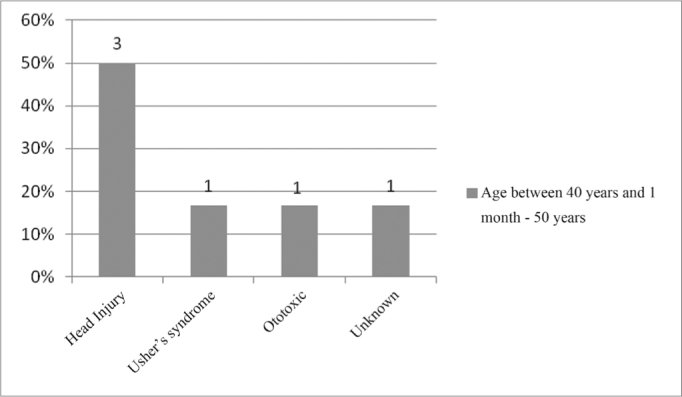
Implanted patient's etiology distribution in the age range between 40 years and 1 month and 50 years.

In the age range between 40 years and 1 month and 50 years, the most commonly found etiology was Head Injury, making up 50% of the cases, as per depicted on Fig. 6.

**Figure 7:**
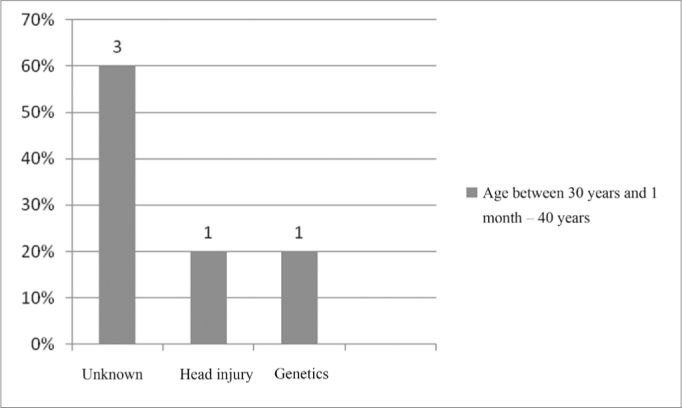
Implanted patient's etiology distribution in the age range between 30 years and 1 month and 40 years.

In the age range between 30 years and 1 month and 40 years, the unknown etiology was the most frequent, as per depicted on Fig. 7.

**Figure 8:**
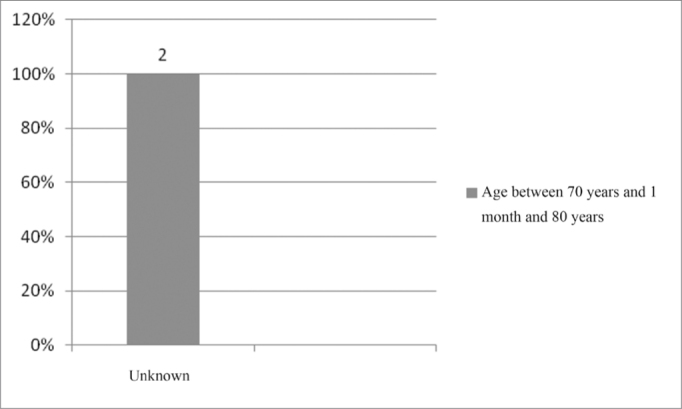
Implanted patient's etiology distribution in the age range between 70 years and 1 month and 80 years.

In the age range between 70 years and 1 month and 80 years, we found 100% of unknown etiology, as per depicted on Fig. 8.

**Figure 9:**
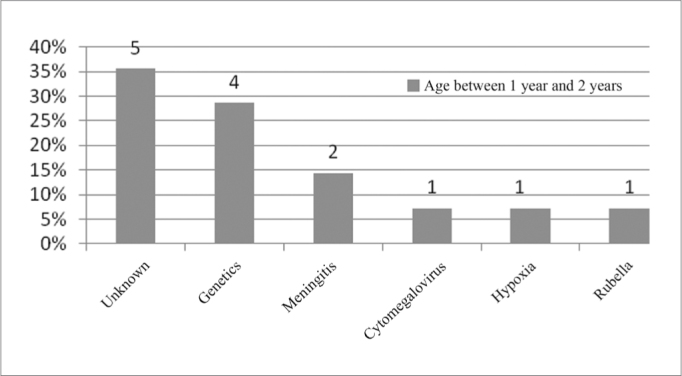
Implanted patient's etiology distribution in the age range between 1 year and 2 years of age.

In the age range between 1 year and 2 years of age, we noticed that the unknown etiology was the most commonly found, as per depicted on Fig. 9.

**Figure 10:**
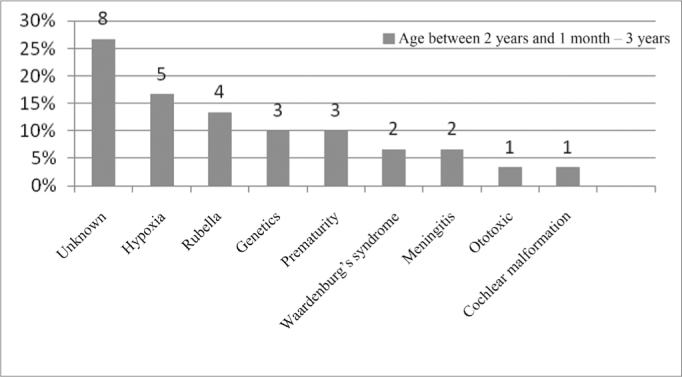
Implanted patient's etiology distribution in the age range between 2 years and 1 month and 3 years.

In the age range between 2 years and 1 month and 3 years, the unknown etiology was the most commonly found, followed by perinatal hypoxia, as per seen in Fig. 10.

**Figure 11:**
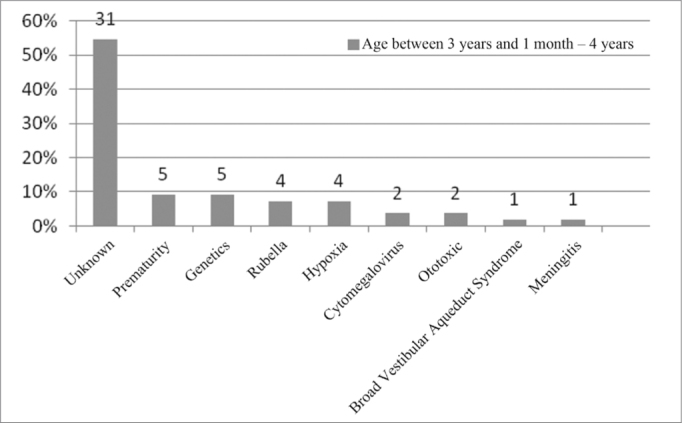
Implanted patient's etiology distribution in the age range between 3 years and 1 month and 4 years.

In the age range between 3 years and 1 month and 4 years, the unknown etiology still is the most commonly found (55%). In this age range, we noticed a higher number of implanted patients, as per described in Fig. 11.

**Figure 12:**
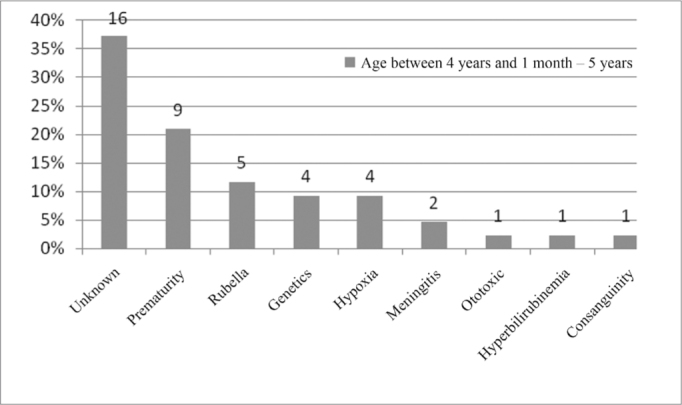
Implanted patient's etiology distribution in the age range between 4 years and 1 month and 5 years.

In the age range between 4 years and 1 month and 5 years, the unknown etiology was the most commonly found, with 37%, followed by prematurity, with 21% as seen in Fig. 12.

**Figure 13:**
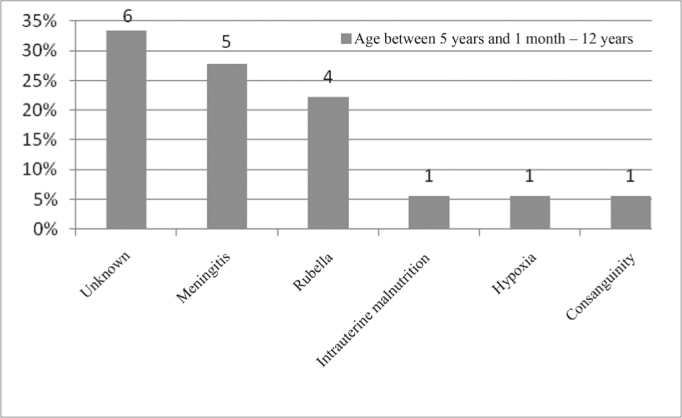
Implanted patient's etiology distribution in the age range between 5 years and 1 month and 12 years.

In the age range between 5 years and 1 month and 12 years, the most commonly found etiology was the unknown, followed by meningitis, as depicted on Fig. 13.

**Figure 14:**
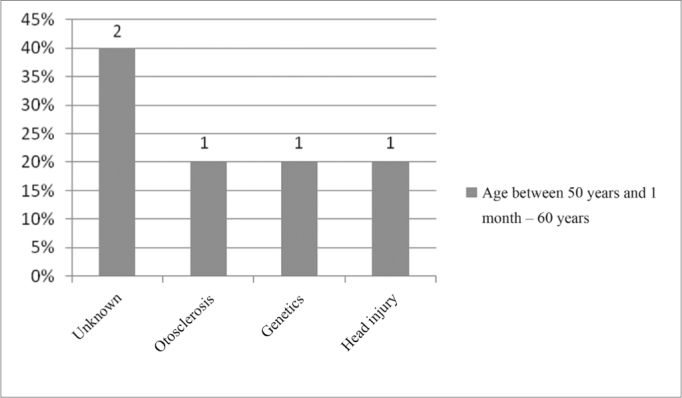
Implanted patient's etiology distribution in the age range between 50 years and 1 month and 60 years.

In the age range between 50 years and 1 month and 60 years, the unknown cause was also the most commonly found, with 40%, followed by otosclerosis, as depicted on Fig. 14.

**Figure 16:**
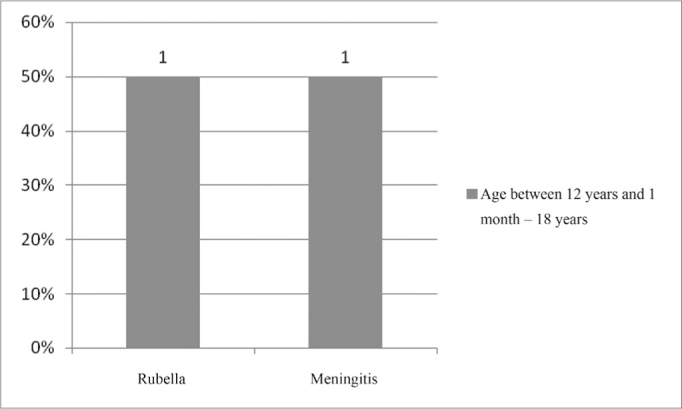
Implanted patient's etiology distribution in the age range between 12 years and 1 month and 18 years.

In the age range between 60 years and 1 months and 70 years, the unknown etiology was also the one most commonly found, in 50% of the cases, followed by genetic cause, ototoxicity and chronic otitis media (COM), as seen in Fig. 16.

**Figure 17:**
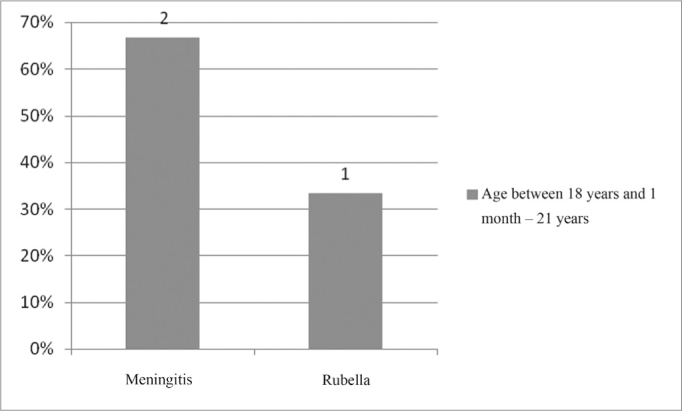
Implanted patient's etiology distribution in the age range between 18 years and 1 month and 21 years.

In the age range between 18 years and 1 month and 21 years, the most commonly found etiology was meningitis, with 67%, according with Figure 17.

## DISCUSSION

In the study we carried out, we analyzed 200 charts from patients enrolled in the cochlear implant program, between August of 2000 and May of 2008, encompassing the age range between 1 year and 73 years (Fig.1), 125 males (62.5%) and 75 females (37.5%), which is in agreement with the findings from national and international studies^1^, ^2^, ^3^ (Fig. 2).

The individuals came from all the regions in the country, representing 19 states (Fig. 3). The etiological diagnosis was obtained by means of an interview carried out with the parents, family members and the patients in cases of young adults and elderly patients.

We approached relevant issues such as: problems during pregnancy, until delivery and post-partum, hereditary factors or factors associated with the current disease. Among the 200 charts studied, we found 19 etiologies of hearing loss (Fig. 4).

Even with a careful interview, the most commonly found cause in our study was the unknown, with 40% among the 200 charts, corroborating prior studies: 44%^4^; 40.7%^2^; 37.5%^5^; 37.31%^6^; 36.6%^1^; 34.3%^7^; 32%^8^ and 31.9%^9^.

The second most found factor was maternal rubella, with 11%, different from the findings of the aforementioned studies, in which maternal rubella had different positions, except in the study^2^ and in the research^10^ carried out in São Caetano do Sul, in a specialized school for the hearing impaired.

In this case, we noticed the same order as the one found in the present study concerning incidence: the unknown etiology in first place and maternal rubella in second. In third place we found genetics - in 10% of the charts analyzed. In all of them there were other cases reported of hearing loss in the family, and such finding is different from papers^1,^^6,^^8,^^9^ in which genetics was the second most commonly found cause.

Prematurity (which is a multifactorial situation) was the fourth most commonly found etiology in our study, with 9%, matching the study^11^ carried out in Salvador, in a special institution for hearing impaired individuals.

Bacterial meningitis was the fifth most commonly found etiology, accounting for 7.5%. However, as observed in previous studies^1,^^8,^^12,^^13^, among the acquired causes, this is the one with the most number of cases.

Perinatal hypoxia was the sixth most commonly found etiology, accounting for 7.5% of the cases, as reported in many national studies^2,^^8,^^12,^^14^. Nonetheless, in some foreign studies^9,^^7,^^13^, it stands out as the third most commonly found cause, and in one of them it shows up in second place insofar as incidence is concerned.

The use of ototoxic drugs came in seventh, accounting for 4%, which is equal to findings from some studies^6,^^8^.

In eighth place came head injury (HI), with 3.5%, which in our study it represented the most commonly found etiology in the age range between 21 years and 1 month and 30 years of age, accounting for 40% (Fig. 5), and in the age range between 40 years and 1 month and 50 years, accounting for 50% of the cases (Fig. 6).

In 9th, came cytomegalovirus - 1.5%, which number of cases overshadows the constant in some studies^7,^^8,^^10^ and it remains short of what was found in one study^13^.

In the age range between 30 years and 1 month and 40 years, the unknown etiology was the one most commonly found - 60% of the cases (Figure 7).

In the age range between 70 years and 1 month and 80 years, we found the unknown etiology with 100% of the cases (Fig. 8). Of the 200 medical charts studied, we found 1% of patients with Waardenburg syndrome and 1% with the Broad Vestibular Aqueduct Syndrome. Other etiologies were found, in 0.5%, such as: cochlear malformation, mumps (lower than the number of cases found in the study^10^), Usher's syndrome, otosclerosis, chronic otitis media (COM), hyperbilirubinemia (much lower than what was found in one study^10^), consanguinity (2.5% in a specific study^10^) and intrauterine malnutrition.

**Figure 15:**
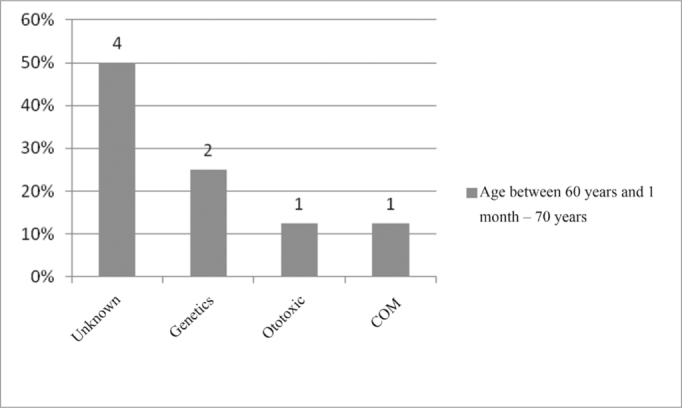
Implanted patient's etiology distribution in the age range between 60 years and 1 month and 70 years.

When we analyzed etiology according to age range, we noticed that the unknown etiology was the one most commonly found in the age range between 1 year and 12 years of age, according to figures 9, 10, 11, 12, 13, 14, 15.

In the age range between 12 years and 1 month and 18 years, there was no predominance of the pathologies found (Fig. 16). Nonetheless, in the age range between 18 years and 1 month and 21 years, the most commonly found pathology was meningitis, in 67% (Fig. 17).

We observed that, in young adults and in elderly individuals, the most commonly found etiologies were the acquired ones.

## CONCLUSION

According to the results obtained, we can state that the unknown etiology continues prevailing, which points to the need of carrying out genetic studies, in cases of congenital sensorineural hearing loss without an apparent cause, with the goal of reaching a real etiologic profile.

Rubella was the second most commonly found cause, and for this etiology there already are preventive measures, similarly to what we have for meningitis. Even then, the incidences of these diseases are still high. Given that, we suggest studies which approach the knowledge, access and effectiveness of these preventive actions.

In the correlation between different etiologies and age ranges, we found varied etiologies, especially when comparing young adults and children, and adults and the elderly. As we can see in some age ranges, life habits influence this result. In young adults, for instance, head injury was the one most commonly found.
